# Adipokine Pattern in Subjects with Impaired Fasting Glucose and Impaired Glucose Tolerance in Comparison to Normal Glucose Tolerance and Diabetes

**DOI:** 10.1371/journal.pone.0013911

**Published:** 2010-11-09

**Authors:** Anke Tönjes, Mathias Fasshauer, Jürgen Kratzsch, Michael Stumvoll, Matthias Blüher

**Affiliations:** 1 Department of Medicine, University of Leipzig, Leipzig, Germany; 2 Institute of Laboratory Medicine, Clinical Chemistry and Molecular Diagnostics, University of Leipzig, Leipzig, Germany; University of Bremen, Germany

## Abstract

**Aim:**

Altered adipokine serum concentrations early reflect impaired adipose tissue function in obese patients with type 2 diabetes (T2D). It is not entirely clear whether these adipokine alterations are already present in prediabetic states and so far there is no comprehensive adipokine panel available. Therefore, the aim of this study was to assess distinct adipokine profiles in patients with normal glucose tolerance (NGT), impaired fasting glucose (IFG), impaired glucose tolerance (IGT) or T2D.

**Methods:**

Based on 75 g oral glucose tolerance tests, 124 individuals were divided into groups of IFG (n = 35), IGT (n = 45), or NGT (n = 43). Furthermore, 56 subjects with T2D were included. Serum concentrations of adiponectin, chemerin, fetuin-A, leptin, interleukin (IL)-6, retinol-binding protein 4 (RBP4), monocyte chemoattractant protein (MCP)-1, vaspin, progranulin, and soluble leptin receptor (sOBR) were measured by ELISAs.

**Results:**

Chemerin, progranulin, fetuin-A, and RBP4, IL-6, adiponectin and leptin serum concentrations were differentially regulated among the four investigated groups but only circulating chemerin was significantly different in patients with IGT compared to those with IFG. Compared to T2D the IFG subjects had higher serum chemerin, progranulin, fetuin-A and RBP4 levels which was not detectable in the comparison of the T2D and IGT group.

**Conclusion:**

Alterations in adipokine serum concentrations are already detectable in prediabetic states, mainly for chemerin, and may reflect adipose tissue dysfunction as an early pathogenetic event in T2D development. In addition, distinct adipokine serum patterns in individuals with IFG and IGT suggest a specific role of adipose tissue in the pathogenesis of these prediabetic states.

## Introduction

Adipose tissue dysfunction belongs to the primary defects in obesity and may link obesity to several health problems including increased risk of type 2 diabetes, fatty liver, and cardiovascular disease [1]–[6]. Altered adipokine serum concentrations are an early symptom of impaired adipose tissue function and may contribute to the development of obesity-associated disorders. In patients with type 2 diabetes, elevated tumor necrosis factor α (TNFα), C-reactive protein (CRP), interleukin (IL) -1, -6, -8, plasminogen activator inhibitor-1 (PAI-1), retinol binding protein 4 (RBP4), chemerin, fetuin-A, visfatin/Nampt, resistin and reduced adiponectin and IL-10 serum concentrations have been reported [7]–[11], reviewed in [1], [5]. However, it is not entirely clear whether these alterations in adipokine serum concentrations are already present in prediabetic states of isolated impaired fasting glycemia (IFG), isolated impaired glucose tolerance (IGT), or combined IFG/IGT. Furthermore, at present there is no comprehensive comparison of adipokine pattern across all stages of glucose intolerance starting from NGT status available. Here, we sought to identify adipokines, which are either increased or decreased in individuals with the prediabetic states IFG and IGT compared to normal glucose tolerant (NGT) healthy controls and subjects with type 2 diabetes. In addition, we tested the hypothesis that isolated IFG and IGT are associated with distinct adipokine patterns which reflect the pathophysiological differences between IFG and IGT. Taking into account the dominant effect of obesity, age and gender on adipokine levels we present all analyses adjusted for these factors. Key defects in IFG include reduced hepatic insulin sensitivity, beta cell dysfunction, and/or chronic low beta cell mass, altered glucagon-like peptide-1 secretion, and inappropriately elevated glucagon secretion. In contrast, IGT is characterised by reduced peripheral insulin sensitivity, near-normal hepatic insulin sensitivity, progressive loss of beta cell function, reduced secretion of glucose-dependent insulinotropic polypeptide, and inappropriately elevated glucagon secretion [12], [13]. In addition, the aetiologies of IFG and IGT seem to differ, with IFG being predominantly related to genetic factors, smoking, and male gender, while IGT is predominantly related to physical inactivity, unhealthy diet, and short stature [13]. Our aim is to elucidate distinct pathomechanisms for either IFG or IGT by comparing adipokine serum patterns between these prediabetic states and NGT and T2D. Furthermore, we aim to provide a broad overview of adipokine profiles across all stages of glucose intolerance.

## Methods

### Subjects

A total of 864 Caucasian men (n = 413) and women (n = 451) have been consecutively recruited in the context of a study on insulin resistance at the Department of Medicine, University of Leipzig, to represent a wide range of obesity, insulin sensitivity, and glucose tolerance. On the basis of a 75-g oral glucose tolerance test (OGTT) according to the criteria of the American Diabetes Association [14], 179 individuals for whom complete data sets for the described parameters were available, were selected from this cohort and divided into groups of isolated IFG (n = 35), isolated IGT (n = 445), NGT (n = 453), T2D (n = 56). Individuals with NGT were defined by a fasting plasma glucose <6.0 mmol/l and a 120-min plasma glucose <7.8 mmol/l, patients with isolated IGT by a fasting plasma glucose <6.0 mmol/l and a 120-min plasma glucose >7.8 mmol/l and <11.1 mmol/l. Isolated IFG was defined by a fasting plasma glucose ≥6.1 mmol/l and ≤7.0 mmol/l as well as a 120-min plasma glucose <7.8 mmol/l following WHO criteria [15]. T2D was defined by 120-min glucose ≥11.1 mmol/l. All individuals fulfilled the following exclusion criteria: 1) Any acute or chronic inflammatory disease as determined by a leucocyte count >7000 Gpt/l, CRP >5.0 mg/dl, or clinical signs of infection; 2) antibodies against glutamic acid decarboxylase (GAD); 3) history of hypertension or systolic blood pressure (SBP) >140 mmHg and diastolic blood pressure (DBP) >85 mmHg; 4) clinical evidence of either cardiovascular or peripheral artery disease; 5) thyroid dysfunction; 6) alcohol or drug abuse; 7) pregnancy. All study protocols have been approved by the ethics committee of the University of Leipzig. All participants gave written informed consent before taking part in the study.

### Measurement of body fat content, glucose metabolism, and insulin sensitivity

Percentage body fat was measured by dual X-ray absorptiometry (DEXA). The OGTT was performed after an overnight fast with 75-g standardized glucose solution (Glucodex Solution 75 g; Merieux, Montreal, Canada). All baseline blood samples were collected between 8:00 and 10:00 am after an overnight fast. Plasma insulin was measured with an enzyme immunometric assay for the IMMULITE automated analyzer (Diagnostic Products, Los Angeles, CA). Serum high-sensitive CRP, and IL-6 were measured as previously described [16].

Insulin sensitivity was assessed using the HOMA-IR as dscribed [17] and in a subgroup (n = 51) with the euglycemic-hyperinsulinemic clamp method using a previously described protocol [18]. Glucose infusion rate (GIR) was calculated from the last 45 min of the clamp, in which glucose infusion rate could be kept constant in order to achieve the target plasma glucose concentration of 5.5 (±5%) mmol/L.

### Measurement of adipokine serum concentrations

All baseline blood samples were collected between 8:00 and 10:00 am after an overnight fast. Serum concentrations of all adipokines have been measured in triplicate and 10 replicates per ELISA plate have been used as internal quality controls. Serum adiponectin was measured using an ELISA (AdipoGen; Seoul, Korea; reference range: 8.1–19.5 µg/ml inter-assay variability: 5.1%; intra-assay variability: 4.4%). Serum leptin was measured using an ELISA (Mediagnost, Reutlingen, Germany; sensitivity: 0.2 ng/ml; reference range of normal leptin values at a BMI of 25 kg/m^2^: males: 1.2–8.9 ng/ml, females: 8–24 ng/ml; inter- and intra-assay variability: <7%). Serum RBP4 was measured by ELISA (AdipoGen; Seoul, Korea;). Percent coefficient of variation for intra- and inter-assay replicate samples was less than 9% for RBP4. Serum MCP-1 concentrations were measured by an immunoassay system (Quantikine human MCP-1 Immunoassay; R&D Systems, Minneapolis, MN) (inter assay variability: <10%; intra-assay variability: <8%). Serum vaspin was measured using a previously desribed ELISA [19] (inter-assay variability: 8.7%; intra-assay variability: <5%). Serum progranulin was measured using a previously developed ELISA [20] (inter-assay variability: 8.5%; intra-assay variability: 6.9%). Serum soluble leptin receptor (sOBR) was measured as previously described [21] (inter- and intra-assay variability: <11.7%). Serum chemerin was measured by ELISA (Biovendor, Heidelberg, Germany) (inter-assay variability: <10%; intra-assay variability: 5.1%). Serum fetuin-A was measured by ELISA (Biovendor, Heidelberg, Germany) (inter- and intra-assay variability: <8%).

### Statistical analyses

Data are shown as means ± SD unless stated otherwise. Before statistical analysis, non-normally distributed parameters were logarithmically transformed to approximate a normal distribution. Overall group differences were assessed by ANOVA with and without adjustment for age, gender, and body mass index (BMI). In order to adjust for age, gender, BMI a general linear model analysis was performed for each adipokine. Non-standardizes residuals were then taken forward as a new dependent variable in the ANOVA analysis for all four groups (NGT, IFG, IGT, T2D). For p-values <0.05 in the ANOVA adjusted for these co-variates a Tukey-HSD posthoc test was performed. Statistical analysis was performed using SPSS version 15.0 (Chicago, IL). Additionally, we performed a second analog analysis but adjusted with waist-to-hip-ratio (WHR) and waist circumference instead of BMI to assess possible effects of fat distribution.

### Power calculation

Based on the distribution of the parameters in our study, we would be able to detect a mean difference of ±20% in serum adipokine levels with a power of 17% to 100% (vaspin 17%, leptin 21%, IL-6 28%, adiponectin 35%, RBP4 46%, MCP-1 99%, chemerin 99%, progranulin 100%, fetuin-A 100%) at a significance level of 0.05 with a sample size of at least 35 subjects per group.

## Results

A total of 179 men (n = 74) and women (n = 105) with an age range from 18 to 77 years and BMI from 21 to 62 kg/m^2^ were studied in groups of NGT, IFG, IGT and T2D (table 1). While age distribution was comparable BMI, waist-to-hip-ratio (WHR), waist circumference, HbA1c, hsCRP and parameters of insulinsensitivity (HOMA-IR and whole body glucose uptake) was significantly different in the comparison of all four groups (table 1). Gender effects were only detectable for serum concentrations of chemerin, adiponectin and leptin (data not shown). BMI had significant impact on progranulin, RBP4, adiponectin and leptin levels (table S1). Moreover, progranulin, fetuin-A, RBP4, IL-6 and leptin were significantly correlated increased waist circumference and adiponectin with decreased waist circumference (table 2). In contrast, chemerin was not correlated to waist or hip circumference but hsCRP (p<0.001, r = 0.262) and HbA1c (p<0.001, r = 0.294, table 2). Chemerin, progranulin, fetuin-A, RBP4, IL-6, leptin and adiponectin serum concentrations significantly correlated with HOMA-IR (independent from BMI, age and gender effects). Whereas higher HOMA-IR was associated with elevated concentration of chemerin (p = 0.022; beta = 0.009), progranulin (p<0.001; beta = 0.016), fetuin-A (p<0.001; beta = 0.012), RBP4 (p<0.001; beta = 0.049), IL-6 (p = 0.012; beta = 0.049) and leptin (p<0.001; beta = 0.031) it was negatively correlated with adiponectin serum levels (p = 0.002; beta = −0.028). In the subgroup of non-diabetic subjects the effects remained significant for progranulin, fetuin-A, RBP4, adiponectin and showed a trend with a consistent effect direction for leptin (table S1).

**Table 1:** Characteristics of the study population.

	NGT	IFG	IGT	T2D	p-value
					(ANOVA)
	*mean (+-SD)*	*mean (+-SD)*	*mean (+-SD)*	*mean (+-SD)*	
	*[range]*	*[range]*	*[range]*	*[range]*	
**N (m/f)**	43 (20/23)	35 (13/22)	45 (17/28)	56 (24/32)	
**Age**	61.3 (±9.3)	61.9 (±12.3)	63.3 (±8.8)	62.4 (±7.2)	0.78
	[44–76]	[20–80]	[40–77]	[45–78]	
**BMI (kg/m2)**	29.6 (±6.3)	33.4 (±7.6)	35.2 (±8.2)	33.9 (±4.1)	0.001
	[21.3–52.7]	[23.4–55.5]	[25.1–62.0]	[26.8–46.5]	
**Body fat (%)**	26.1 (±8.1)	31.2 (±9.6)	34.5 (±9.5)	30.0 (±4.1)	<0.001
	[12.5–49.8)	[16.2–52.7]	[22.2–59.8]	[22.9–42.1]	
**fasting glucose (mmol/l)**	5.01 (±0.45)	7.11 (±0.69)	5.60 (±0.37)	6.56 (±0.91)	<0.001
	[3.59–5.85)	[6.25–8.61]	[4.52–6.03]	[5.24–9.39]	
**2-hr glucose (mmol/l)**	5.97 (±0.75)	5.83 (±0.60)	8.9 (±0.83)	NA	<0.001
	[4.65–7.32]	[4.44–7.21]	[7.84–10.94]	NA	
**HbA1c (%)**	5.42 (±0.23)	5.59 (±0.26)	5.92 (±0.19)	6.4 (±0.36)	<0.001
	[5.1–6.1]	[5.3–6.5]	[5.6–6.3]	[5.8–7.4]	
**fasting insulin (pmol/l)**	62.9 (±63.9)	87.6 (±63.0)	152 (±103)	197.1 (±97.5)	<0.001
	[6–351]	[17–347]	[25–411]	[71–504]	
**hsCRP (mg/dl)**	0.52 (±0.81)	0.53 (±0.81)	0.85 (±0.73)	1.19 (±0.78)	<0.001
	[0.01–4.31]	[0.02–4.19]	[0.05–3.17]	[0.13–3.64]	
**Free fatty acids (mmol/l)**	0.34 (±0.21)	0.60 (±0.34)	0.63 (±0.28)	0.91 (±0.31)	<0.001
	[0.05–0.94]	[0.18–1.98]	[0.13–1.34]	[0.19–1.43]	
**Waist circumference (cm)**	95.2 (±18.2)	106.0 (±19.9)	112.3 (±16.8)	114.4 (±13.6)	<0.001
	[58–135]	[65–150]	[75–156]	[65–144]	
**Hip circumference (cm)**	107.5 (±18.3)	115.9 (±19.4)	116.6 (±18.6)	114.6 (±10.5)	0.046
	[81–184]	[88–193]	[96–177]	[94–135]	
**WHR**	0.89 (±0.13)	0.92 (±0.13)	0.97 (±0.14)	1.0 (±0.13)	<0.001
	[0.64–1.16]	[0.64–1.26]	[0.69–1.26]	[0.69–1.34]	
**HOMA_IR**	2.1 (±2.2)	4.0 (±2.9)	5.45 (±3.70)	8.26 (±4.3)	<0.001
	[0.2–11.9]	[0.7–16]	[1.0–14.9]	[3.1–26.2]	
**whole body glucose uptake**	86.5 (±26.2)	63.3 (±34.7)	75.7 (±36.4)	29.7 (±18.9)	<0.001
**(µmol/kg/min),**	[23–119]	[9–98]	[10–119]	[8–62]	
	(N = 22)	(N = 7)	(N = 12)	(N = 10)	

Group specific arithmetic means, standard deviation and ranges are provided. Furthermore, p-values for the univariate ANOVA are given to assess significant differences between the groups.

**Table 2:** Univariate correlation of adipokine concentrations with anthropometric parameters, CRP levels and HbA1c.

	waist [cm]		hip [cm]		body fat [%]		hsCRP [mg/dl]		HbA1c [%]	
Adipokine	p-value	r	p-value	r	p-value	r	p-value	r	p-value	r
Chemerin [ng/ml]	0.293	0.079	0.187	0.099	0.294	0.079	***<0.001***	0.262	***<0.001***	0.294
Progranulin [ng/ml]	***<0.001***	0.400	***<0.001***	0.263	***<0.001***	0.316	***<0.001***	0.488	***<0.001***	0.387
Fetuin-A [µg/ml]	***<0.001***	0.268	***<0.001***	0.266	***0.003***	0.219	***0.005***	0.210	***<0.001***	0.348
RBP4 [µg/ml]	***<0.001***	0.394	***<0.001***	0.299	***<0.001***	0.350	***<0.001***	0.374	***<0.001***	0.391
IL-6 [pg/ml]	***0.002***	0.230	0.057	0.143	***0.017***	0.179	***<0.001***	0.319	***<0.001***	0.309
Adiponectin [µg/ml]	***<0.001***	−0.476	0.072	−0.135	0.090	−0.127	***0.026***	−0.166	***<0.001***	−0.293
sOBR [ng/ml]	0.379	−0.066	0.866	0.011	0.822	−0.017	0.081	0.131	0.277	0.082
Vaspin [ng/ml]	0.928	−0.007	0.666	0.033	0.217	0.093	0.570	0.043	0.425	0.060
Leptin [ng/ml]	***<0.001***	0.355	***<0.001***	0.732	***<0.001***	0.675	***<0.001***	0.410	***0.031***	0.162
MCP-1 [pg/ml]	0.408	0.062	0.396	−0.064	0.630	−0.036	0.174	−0.102	0.220	0.092

Data present p-values and Pearson correlation coefficients assessed by univariate correlation analysis in all 179 subjects. P-values <0.05 were considered as nominal significant and marked in bold italic.

Consistently with published data there were significant differences in the overall comparison of all four groups in the serum levels detectable for progranulin, fetuin-A, RBP4, IL-6, adiponectin and leptin. All differences remained significant after adjusting for age, gender and BMI. In contrast, MCP-1, vaspin and sOBR did not show significantly altered serum levels between NGT, IFG, IGT or T2D (table 3). Compared to NGT controls, adiponectin serum concentrations were significantly lower in individuals with IGT (p = 0.008). Patients with IGT had significantly higher progranulin (p<0.004), RBP4 (p = 0.010), and fetuin-A (p = 0.001) serum concentrations than individuals in the NGT group. As a key result of our study, we found distinct adipokine patterns in individuals with isolated IFG and isolated IGT independent from BMI. Individuals with IGT and T2D had significantly higher chemerin, progranulin, fetuin-A, and RBP4 serum concentrations than IFG and NGT subjects (table 3). However, only differences in circulating chemerin between the IFG and IGT group remained significant after adjusting for age, gender, and BMI (p = 0.002) (table 3). There were no significant differences in adiponectin, leptin, sOBR, vaspin, MCP-1, and IL-6 serum concentrations between the IGT and IFG groups. However, the post hoc test including all groups revealed that the adipokine serum profile in IFG subjects showed more similarities with the NGT group and IGT subjects overlap in a greater extend with the T2D group (table 3). There were no significant differences detectable between the NGT and IFG group after adjusting for age, sex and BMI and only circulating IL-6 and leptin varied between T2D and IGT subjects (table 3).

**Table 3:** Adipokine serum concentrations in individuals with normal glucose tolerance (NGT), isolated impaired fasting glucose (IFG), impaired glucose tolerance (IGT) and type 2 diabetes (T2D).

					p-value	p-value adj	p-value Tukey-HSD					
	NGT	IFG	IGT	T2D	(ANOVA)	(ANOVA)	NGT vs IFG	NGT vs IGT	IFG vs IGT	NGT vs T2D	IFG vs T2D	IGT vs T2D
**Chemerin (ng/ml)**	197.4 (±38.7)	193.3 (±36.9)	227.3 (±42.5)	237.9 (±50.2)	***<0.001***	***<0.001***	0.828	***0.016***	***0.002***	***<0.001***	***<0.001***	0.567
	[113–323]	[131–324]	[166.7–366.9]	[155.2–380.2]								
** Progranulin (ng/ml)**	138.8 (±24.9)	155.4 (±31.3)	178.2 (±40.0)	192.2 (±53.7)	***<0.001***	***<0.001***	0.693	***0.004***	0.140	***<0.001***	***<0.001***	0.227
	[98–205]	[106–239]	[95–257]	[104–319]								
**Fetuin-A (µg/ml)**	275.4 (±43.5)	299.8 (±46.2)	324.2 (±51.5)	334.6 (±50.8)	***<0.001***	***<0.001***	0.305	***0.001***	0.249	***<0.001***	***0.011***	0.574
	[210–399]	[217–419]	[214–398]	[205–421]								
**RBP4 (µg/ml)**	49.2 (±22.1)	62.9 (±28.4)	70.8 (±23.6)	78.2 (±24.1)	***<0.001***	***<0.001***	0.279	***0.010***	0.635	***<0.001***	***0.007***	0.135
	[13.6–106.7]	[23.4–152.1]	[26.6–112.4]	[42.6–143.8]								
**IL-6 (pg/ml)**	2.15 (±1.31)	2.88 (±2.73)	2.68 (±4.16)	5.0 (±2.87)	***<0.001***	***<0.001***	0.963	**0.096**	0.317	***<0.001***	***<0.001***	***<0.001***
	[0.3–5.9]	[0.1–11.5]	[0.04–27.0]	[1.7–17.3]								
**Adiponektin (µg/ml)**	8.8 (±4.7)	7.2 (±4.7)	6.2 (±3.2)	5.8 (±2.8)	***0.002***	***0.001***	0.088	***0.008***	0.908	***0.001***	**0.603**	0.930
	[2.6–23.7]	[1.9–23.2]	[1.3–13.3]	[1.4–10.9]								
**Leptin (ng/ml)**	16.6 (±12.0)	21.9 (±16.9)	22.3(±16.4)	25.3 (±10.7)	***0.001***	***<0.001***	0.994	0.802	0.935	***<0.001***	***<0.001***	***<0.001***
	[3.0–61.3]	[3.5–71.9]	[3.2–72.9]	[8.4–53.4]								
**sOBR (ng/ml)**	27.3 (±7.1)	28.7 (±9.9)	27.8 (±6.2)	29.6 (±7.5)	0.441							
	[15.0–44.6]	[15.0–62.3]	[15.6–43.7]	[15.6–55.9]								
**Vaspin (ng/ml)**	1.55 (±1.31)	2.01 (±1.77)	1.89 (±1.76)	2.07 (±1.45)	0.179							
	[0.1–5.2]	[0.1–6.8]	[<0.1–7.8]	[0.1–6.8]								
**MCP-1 (pg/ml)**	398.5 (±78.4)	384.6 (±73.2)	398.5 (±98.4)	405.2 (±106.6)	0.912							
	[237–561]	[259–526]	[246–624]	[206–657]								

Data present means ± SD. P-values in the adjusted ANOVA are corrected for age, gender and BMI. Tukey-HSD post-hoc test was performed only when the unadjusted ANOVA showed significant differences.

When adjusting for waist circumference instead of BMI or WHR out of all comparisons between the groups only one result switched from non significant to nominal significant (difference of IGF group to IGT group for progranulin levels, see table S2). There were no substantial differences between adjustment with BMI compared to WHR or waist circumference for comparisons between NGT and IFG as well as NGT and IGT subjects. The results for comparisons of NGT subjects to the T2D group remained unaltered by adjustement with WHR or waist circumference instead of BMI for all adipokines apart from leptin. The significant difference in serum leptin concentrations diminished by adjustment with waist circumference (table S2). Furthermore, the differences in serum leptin between the IGT and T2D group were decreased after adjustment with WHR and waist circumference instead of BMI (table S2).

## Discussion

It has been recognized that altered circulating adipokine patterns can be early abnormalities in obesity and may contribute to obesity-related diseases including impaired glucose metabolism and development of type 2 diabetes [22]. This study provides new insight into adipokine-related pathomechanisms for the development of prediabetic states including isolated IFG and isolated IGT. It has been suggested that distinct pathomechanisms underly the IFG and IGT phenotypes [13]. Hepatic insulin resistance, stationary beta cell dysfunction, chronic low beta cell mass, and others are causative factors in the pathogenesis of IFG, whereas IGT is characterized by reduced peripheral insulin sensitivity, near-normal hepatic insulin sensitivity, and progressive loss of beta cell function [13]. However, the role of adipose tissue dysfunction and altered adipokine serum concentrations in these different entities of prediabetic states is not well defined.

Firstly, we confirmed previous reports (reviewed in [22]) that prediabetic states (IFG, IGT or both) are associated with increased progranulin, chemerin, fetuin-A, RBP4, and decreased adiponectin serum concentrations compared to individuals with NGT. The distinct adipokine serum patterns which we observed in individuals with IFG and IGT suggest a specific role of adipose tissue in the pathogenesis of these prediabetic states. Among nine different adipokines, circulating chemerin, progranulin, fetuin-A, and RBP4 serum concentrations were significantly higher in individuals with IGT compared to those with IFG. Increased circulating concentrations of chemerin [23], progranulin [20], RBP4 [8], and fetuin-A [24] have been shown to be associated with insulin resistance. Therefore, our results support the view that peripheral insulin resistance may represent a distinct pathology in IGT but not in IFG development. However, only higher chemerin serum concentrations were significantly associated with IGT after adjustment for age, gender, and BMI. This suggests that elevated RBP4 and fetuin-A serum levels do not represent an independent mechanism linking adipose tissue dysfunction to impaired glucose metabolism, whereas chemerin may be closely reflect causation in obesity-related glucose intolerance. However, we can not entirely exclude the possibility that statistical power was not sufficient to detect age, gender, and BMI-independent differences in fetuin-A, progranulin and RBP4 serum concentrations between the IGT and IFG groups. It has been previously shown that fasting glucose regulation is more related to abdominal obesity (waist circumference), whereas 2 h glucose regulation is more associated with overall degree of obesity [25], [26]. Among the different adipokines, only chemerin and progranulin serum concentration significantly (and independently) predicted the difference between IFG and IGT, suggesting that chemerin may be an additional predictor of abdominal obesity in prediabetic state. We tested whether adjusting for waist circumference or WHR would have a significant effect on the observed significant group differences in adipokine serum concentrations. Interestingly, adjusting for waist circumference instead of BMI or WHR only affected the difference in circulating progranulin between IFG and IGT group. Since progranulin serum concentrations are both related to visceral and whole body fat mass [20], this parameter could not be used to dissect a pathophysiology of glucose metabolism in the fasted versus postprandial state. In addition, significant group difference in serum leptin concentrations were abolished by adjustment for waist circumference. There were no additional substantial differences between adjustment for BMI, waist circumference or WHR for a better prediction of the glucose tolerance category.

We recently reported that elevated progranulin serum concentrations were associated with visceral obesity, elevated plasma glucose, and dyslipidemia [20]. We identified progranulin as a novel marker of chronic inflammation in obesity and type 2 diabetes which closely reflects omental adipose tissue macrophage infiltration [20]. Therefore, elevated progranulin serum concentrations in IGT compared to IFG patients may suggest that inflammation of adipose tissue contributes to the development of IGT but not of IFG. Further studies are necessary to elucidate such potential differences in adipose tissue morphology between individuals with IGT and IFG.

Chemerin is highly expressed in liver and adipose tissue and is involved in anti-inflammatory pathways in activated macrophages [23]. Chemerin is known to be associated with a range of markers of the metabolic syndrome [27]. Recently, chemerin was shown to be increased by hyperinsulinaemia in women with PCOS [28]. Moreover, changes in HOMA-IR under metformin treatment significantly predict changes in serum chemerin [28]. Our results support these findings since significantly higher circulating chemerin in individuals with IGT was associated with significantly higher fasting insulin serum concentrations in patients with IGT compared to those with IFG.

Since false negative associations due to the limited sample size should be taken into account especially for vaspin, leptin, IL-6, adiponectin and RBP4 further studies with extended sample size are required to elucidate the relative role of the different adipokines in relation to deterioration of glucose metabolism from IFG to IGT and ultimately T2D.

In conclusion, alterations in adipokine serum concentrations are already detectable in prediabetic states and may reflect adipose tissue dysfunction as an early pathogenic event in type 2 diabetes development. In addition, higher chemerin and progranulin serum concentrations in the IGT compared to the IFG group suggest a specific role of adipose tissue in the pathogenesis of IGT, but not IFG.

## Supporting Information

Table S1 Effects of HOMA-IR and BMI on adipokine serum concentrations. Results of linear regression analyses for effects of HOMA-IR and BMI on serum adipokine levels in all 179 subjects. Age, gender, BMI and HOMA-IR were included in the model simultaneously.
(0.05 MB DOC)

Table S2 Comparison of adipokine serum concentrations in individuals with normal glucose tolerance (NGT), isolated impaired fasting glucose (IFG), impaired glucose tolerance (IGT) and type 2 diabetes (T2D). P-values are corrected for age, gender and BMI or WHR (light grey line) or waist circumference (dark grey line) respectively. Tukey-HSD post-hoc test was performed only when the unadjusted ANOVA showed significant differences.
(0.06 MB DOC)
