# Supplementary material for: Adipokine Pattern in Subjects with Impaired Fasting Glucose and Impaired Glucose Tolerance in Comparison to Normal Glucose Tolerance and Diabetes
Source: PLoS One. 2010 Nov 9;5(11):e13911. doi: 10.1371/journal.pone.0013911 (PMC2976690; doi:10.1371/journal.pone.0013911)
Supplement: Table S1 — Effects of HOMA-IR and BMI on adipokine serum concentrations. Results of linear regression analyses for effects of HOMA-IR and BMI on serum adipokine levels in all 179 subjects. Age, gender, BMI and HOMA-IR were included in the model simultaneously. (0.05 MB DOC) [file pone.0013911.s001.doc]

|  | **all subjects** | | | | **non-diabetic subjects** | | | |
| --- | --- | --- | --- | --- | --- | --- | --- | --- |
|  | **BMI** | | **HOMA-IR** | | **BMI** | | **HOMA-IR** | |
| **Adipokine** | **p-value** | **beta** | **p-value** | **beta** | **p-value** | **beta** | **p-value** | **beta** |
| Chemerin | 0.820 | 0.001 | ***0.022*** | 0.009 | 0.466 | 0.002 | 0.495 | 0.004 |
| Progranulin | ***<0.001*** | 0.012 | ***<0.001*** | 0.016 | ***0.006*** | 0.008 | ***0.031*** | 0.013 |
| Fetuin-A | 0.158 | 0.003 | ***<0.001*** | 0.012 | 0.163 | 0.003 | ***0.030*** | 0.011 |
| RBP4 | ***<0.001*** | 0.016 | ***<0.001*** | 0.049 | 0.111 | 0.008 | ***<0.001*** | 0.078 |
| IL-6 | 0.145 | 0.018 | ***0.012*** | 0.049 | 0.085 | 0.026 | 0.616 | -0.016 |
| Adiponectin | ***0.007*** | -0.016 | ***0.002*** | -0.028 | 0.058 | -0.013 | ***0.012*** | -0.038 |
| sOBR | 0.916 | <0.001 | 0.542 | -0.003 | 0.800 | -0.001 | 0.445 | -0.006 |
| Vaspin | 0.373 | -0.013 | 0.463 | 0.017 | 0.338 | -0.017 | 0.996 | -0.002 |
| Leptin | ***<0.001*** | 0.044 | ***<0.001*** | 0.031 | ***<0.001*** | 0.045 | 0.055 | 0.026 |
| MCP-1 | 0.990 | <0.001 | 0.813 | -0.001 | 0.965 | <0.001 | 0.414 | 0.005 |
