# Supplementary material for: Adipokine Pattern in Subjects with Impaired Fasting Glucose and Impaired Glucose Tolerance in Comparison to Normal Glucose Tolerance and Diabetes
Source: PLoS One. 2010 Nov 9;5(11):e13911. doi: 10.1371/journal.pone.0013911 (PMC2976690; doi:10.1371/journal.pone.0013911)
Supplement: Table S2 — Comparison of adipokine serum concentrations in individuals with normal glucose tolerance (NGT), isolated impaired fasting glucose (IFG), impaired glucose tolerance (IGT) and type 2 diabetes (T2D). P-values are corrected for age, gender and BMI or WHR (light grey line) or waist circumference (dark grey line) respectively. Tukey-HSD post-hoc test was performed only when the unadjusted ANOVA showed significant differences. (0.06 MB DOC) [file pone.0013911.s002.doc]

|  | **p-value Tukey-HSD** | | | | | |
| --- | --- | --- | --- | --- | --- | --- |
|  | **NGT vs IFG** | **NGT vs IGT** | **IFG vs IGT** | **NGT vs T2D** | **IFG vs T2D** | **IGT vs T2D** |
| **Chemerin (ng/ml)** | 0.828 | ***0.016*** | ***0.002*** | ***<0.001*** | ***<0.001*** | 0.567 |
|  | 0.811 | ***0.031*** | ***0.003*** | ***0.001*** | ***<0.001*** | 0.712 |
|  | 0.724 | ***0.007*** | ***0.001*** | ***0.001*** | ***<0.001*** | 0.666 |
| **Progranulin (ng/ml)** | 0.693 | ***0.004*** | 0.140 | ***<0.001*** | ***<0.001*** | 0.227 |
|  | 0.501 | ***0.003*** | 0.232 | ***<0.001*** | ***0.020*** | 0.746 |
|  | 0.345 | ***<0.001*** | ***0.034*** | ***<0.001*** | ***0.010*** | 0.618 |
| **Fetuin-A (µg/ml)** | 0.305 | ***0.001*** | 0.249 | ***<0.001*** | ***0.011*** | 0.574 |
|  | 0.234 | ***0.001*** | 0.300 | ***<0.001*** | ***0.046*** | 0.829 |
|  | 0.288 | ***0.002*** | 0.182 | ***<0.001*** | ***0.051*** | 0.793 |
| **RBP4 (µg/ml)** | 0.279 | ***0.010*** | 0.635 | ***<0.001*** | ***0.007*** | 0.135 |
|  | 0.160 | ***0.007*** | 0.762 | ***<0.001*** | 0.140 | 0.616 |
|  | 0.213 | ***0.010*** | 0.511 | ***<0.001*** | 0.081 | 0.476 |
| **IL-6 (pg/ml)** | 0.963 | 0.096 | 0.317 | ***<0.001*** | ***<0.001*** | ***<0.001*** |
|  | 0.996 | 0.196 | 0.351 | ***<0.001*** | ***<0.001*** | ***<0.001*** |
|  | 0.968 | 0.191 | 0.337 | ***0.003*** | ***0.001*** | ***<0.001*** |
| **Adiponektin (µg/ml)** | 0.088 | ***0.008*** | 0.908 | ***0.001*** | 0.603 | 0.930 |
|  | 0.063 | ***0.010*** | 0.967 | ***0.007*** | 0.971 | 1 |
|  | 0.172 | ***0.043*** | 0.881 | ***0.027*** | 0.946 | 1 |
| **Leptin (ng/ml)** | 0.994 | 0.802 | 0.935 | ***<0.001*** | ***<0.001*** | ***<0.001*** |
|  | 0.781 | 0.781 | 1 | ***<0.001*** | ***0.028*** | ***0.012*** |
|  | 0.984 | 0.790 | 0.897 | ***0.066*** | ***0.039*** | ***0.003*** |
